# Failures at every level: breakdown of the epigenetic machinery of aging

**DOI:** 10.1093/lifemedi/lnac016

**Published:** 2022-06-28

**Authors:** Dongxin Zhao, Song Chen

**Affiliations:** Shanghai Institute of Materia Medica, Chinese Academy of Sciences, Shanghai 201203, China; Wellcome Sanger Institute, Wellcome Genome Campus, Cambridge CB10 1SA, UK

**Figure 1.**
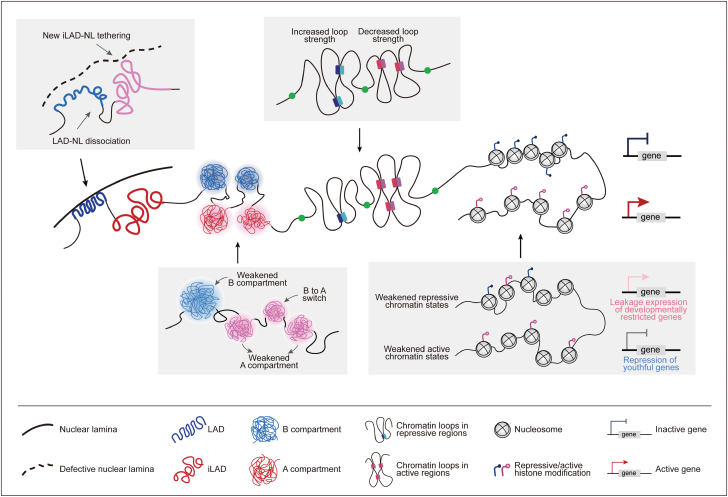
Schematic overview of spatial genome architectures in growing (center) and senescent (gray boxes) stem cells. Senescence is accompanied by erosion of chromatin organization across multiple hierarchic layers, which exhibits convergent epigenetic patterns due to the weakened repressive (shown in blue) and active (shown in red) chromatin states. NL, nuclear lamina.

Aging is a complex and gradual biological process that leads to increased vulnerability to several major human diseases. As global population aging poses significant public health challenges, a deeper understanding of the molecular and cellular basis that drives human aging would be highly valuable in finding solutions to improve physical health and quality of life. Research over the past decades has made unprecedented achievements and reached several hallmarks of aging [1–3]. One such hallmark is epigenetic alterations, which have been shown to drive the aging clock and contribute to aging phenotypes [4, 5]. Epigenetic alterations represent aberrations in the hierarchical organization of the 3D genome. Notably, in metazoan cell nuclei, hundreds of large chromosome domains are positioned at the nuclear lamina, termed lamina-associated domains (LADs). Within LADs and inter-LADs (iLADs), chromosomes are further segregated into two functionally distinct compartments, namely A (active) and B (inactive). On a scale below the compartments, chromatin interactions are predominantly enriched within the insulated self-interacting regions termed topologically associating domains (TADs), which consist of numerous regulatory loops responsible for long-range promoter-enhancer communication. As the lowest level involves the folding of chromatin fibers, chromatin looping correlates with various DNA and histone modifications and participates in transcriptional regulation (Fig. 1). In research of aging, numerous studies have focused on the linear chromatin marks and have uncovered that the loss of heterochromatin, the shifts in the balance of activating and repressive histone modifications, and the decrease in DNA methylation are the characteristics of cellular aging. However, the roles of higher-order genomic organization and the links across epigenetic hierarchical layers in aged cells remain incompletely understood.

Deterioration of the nuclear architecture is a main characteristic of aging [1]. On the inner side of the nuclear envelope, the nuclear lamina meshwork, which is mainly composed of lamins, provides a supporting framework for the nuclear envelope and anchoring sites for interphase chromatin. The decline in ­lamina-associated proteins has been reported as a senescence-associated biomarker in various cellular models [6]. In addition, mutations in ­lamin-encoding genes cause accelerated aging diseases, such as ­Hutchinson-Gilford ­progeria syndrome [7]. A previous study has demonstrated that defects in nuclear lamina lead to dramatic changes in the physical structure of chromatin [8]. However, how the epigenome reprograms hierarchically from nuclear integrity to compartment segregation and further to chromatin modification during the continuous aging process, and how epigenetic alterations determine ­senescence-associated phenotypes and gene signatures, remain to be elucidated.

To address these aspects, Liu et al. recently profiled comprehensive landscapes of chromosome territories, 3D genome structures, and chromatin states to reveal global reorganization within and across epigenomic hierarchical layers over the course of senescence in human mesenchymal progenitor cell models [9]. As age-related decline in tissue homeostatic and regenerative capacities are driven by stem cell degeneration, researchers have deployed a panel of isogenic human mesenchymal progenitor cells as cellular models of senescence and accelerated aging. Upon multimodal data integration, Liu et al. discovered ­aging-associated erosion of chromatin architectures, including the disruption of nuclear positioning, weakening and switching of topological compartments, rewiring of local and distal interactions, redistribution of chromatin states, and an increase in epigenetic entropy (Fig. 1).

At the highest topological level of genome organization, analysis of age-related alterations manifested the overall dissociation of LAD-nuclear lamina, newly formed iLAD-nuclear lamina tethering, and unveiled genomic regions that underwent LAD-to-iLAD switches at the borders. These dramatic changes in genome positioning at the nuclear periphery were highly correlated with aberrant chromatin compartmentalization. The B compartments that were enriched in LADs suffered a ­heterochromatin-loosening state, whereas a small fraction of B compartments within the regions of LAD-to-iLAD switches converted into A compartments.

At the chromatin organization level, although the A/B compartments largely maintained their identities during cellular senescence, Liu et al. showed that up to approximately 20% of genomic regions underwent sub-compartment switches, which is five times higher than those between compartments. Most subcompartment switches restrained within one compartment became less activating or repressing environments. Switches leading to A/B compartment transitions either occurred between weak euchromatin and heterochromatin or were located at the border of A/B compartments, suggesting unstable and interchangeable states in these regions.

At the chromatin interaction level, Liu et al. observed stable TADs with slightly increased boundary strengths during senescence. Closer inspection showed that in young cells, chromatin loops exhibited stronger pairwise interactions in A compartments than in B compartments. Surprisingly, this phenomenon was compromised in aged cells. In addition, loop pairs with either decreased or increased interaction strengths were enriched in active or repressive chromatin states, respectively. Collectively, the consistent findings across multiple hierarchical layers suggest that the overall narrowing of feature differences between heterochromatin and euchromatin is indicative of senescence.

At the chromatin state level, Liu et al. explored the prominent chromatin state transitions represented in each type of higher-order spatial organization switches. Inside LADs, heterochromatin was compromised by DNA hypomethylation and the loss of constitutive histone marks, resulting in the strong-to-weak and weak-to-quiescent repressive state transitions. Interestingly, researchers have also observed a high tendency of transcriptional leakage of developmentally restricted genes and repetitive elements in repressive genomic regions. Within iLADs, active markers and chromatin accessibility displayed global reductions, corresponding to the strong-to-weak and weak-to-quiescent active state transitions. Consequently, genes downregulated in aged cells were predominantly located in the euchromatin state. Lastly, chromatin state transitions that coincided with LAD/iLAD switches mainly occurred in the facultative heterochromatin around iLAD/LAD borders, where the histone modification changes responsible for being quiescent chromatin states or reduced differences between constitutive and facultative heterochromatin occurred.

At the gene expression level, Liu et al. discovered that global activation of the pregnancy-specific beta-1 glycoprotein (*PSG*) gene family is a common feature of physiological, premature, and environmentally induced aging. Interestingly, the transcription of all 11 *PSG* genes was controlled in an insulated neighborhood and organized into one tightly packed TAD/LAD region. The expression of *PSG* genes was deeply repressed in young cells, except for the placenta, but released in aged cells as a consequence of a series of epigenetic reorganizations. Furthermore, the researchers also confirmed the hypothesis that ectopic activation of placenta-specific *PSG* genes in young cells produces senescence-associated phenotypes, such as abolished proliferation ability, which are shared with placenta syncytiotrophoblasts. Collectively, these results demonstrate that the placenta-specific *PSG* gene family serves as a novel biomarker and a potential molecular driver of aging.

In summary, Liu et al. provided an atlas of chromatin landscapes of cellular aging to date the most comprehensive and detailed descriptions of multiple epigenetic hierarchical layers. They highlighted the common and distinct characteristics of physiological and premature aging through systematic investigation of a rich panel of isogenic stem cell models and revealed new mechanisms of epigenetic reorganization in cellular senescence. A remarkable characteristic of senescent cells is the erosion of chromatin organization, which manifests as instability at the borders of insulated genomic neighborhoods and depolarization of repressive and active chromatin states. The authors have referred to this newly reported characteristic as “Convergent Alteration of the Epigenomic Landscape during Aging” (CAEA).

Following the elucidation of the first comprehensive chromatin landscapes of aging, the subsequent requirement is to elucidate how chromosome structure determined function. In particular, querying the functional outcomes of perturbing epigenome is pivotal to distinguishing causal or coincidental effects, and is now feasible with the rapid advances in epigenetic manipulation technologies. The studies of epigenomic drifts should be broadened to include more diverse cell types and physiological situations to interrogate context-dependent mechanisms. Comparisons of epigenome dynamics in aging with those in reversing processes, such as partial reprogramming, can be harnessed to define key checkpoints and trajectories of rejuvenation.

Overall, this study has advanced the comprehensive understanding of epigenomic features during human cellular aging, thus enabling the development of early biomarkers for aging and related diseases and providing a rationale to design potential intervention strategies for aging-related disorders.
